# Secondary Use of Data: Non-Interventional Study Best Practices in Planning and Protocol Development

**DOI:** 10.36469/9796

**Published:** 2017-04-24

**Authors:** Juanzhi Fang, Sara Bruce Wirta, Kristijan Kahler

**Affiliations:** 1 Novartis Pharmaceuticals Corporation, East Hanover, NJ, USA; 2 Novartis Sverige AB, S-183 79 Taby, Sweden

**Keywords:** retrospective study, non-interventional study, secondary use of data, best practices

## Abstract

Well established guidelines already exist that address best practices for Non-Interventional Study (NIS) design and methods. These guidelines provide advice on things to consider while designing a study and developing a protocol, but do not necessarily capture specific details related to the implementation of NIS. The intent of this paper is to propose a best practice for conducting secondary use of data NIS. We propose that the ideal implementation of a NIS should include the development of a strong Study Concept, followed by a detailed Protocol, Analysis Plan, Report, and considerations for Dissemination.

We review and discuss common mistakes/pitfalls and key considerations at each step from concept to publication. In many cases in this review, we have also provided suggestions or accessible resources that researchers can apply as a “best practices” guide when planning, conducting, or reviewing this investigative method.

## INTRODUCTION

There are many synonyms for Non-Interventional Studies (NIS), such as Observational Studies, Real World Studies, Epidemiologic Studies; the term Non-Interventional Studies is now used in regulatory documents in the US and Europe, and appears to be the preferred term for such studies. According to ENCePP (defined in Dir 2001/20/EC Art 2(c)), an NIS is a study where the following requirements are cumulatively fulfilled: (1) the medicinal product is prescribed in the usual manner in accordance with the terms of the marketing authorization, (2) the assignment of the patient to a particular therapeutic strategy is not decided in advance by a trial protocol but falls within current practice and the prescription of the medicine is clearly separated from the decision to include the patient in the study; and (3) no additional diagnostic or monitoring procedures are applied to the patients and epidemiological methods are used for the analysis of collected data.

Non-interventional studies can be segmented by those that involve primary data collection and those that involve secondary use of data (i.e., use of existing data). Primary data collection constitutes any type of study where original data are collected specifically for the purpose of the study and directly from patients (or proxies) and/or health care professionals. Examples include cross-sectional surveys, registries, and studies based on questionnaires capturing patient reported outcomes. Secondary use of data constitutes studies where already existing data are used and all the events of interest have already happened. This may include database research (e.g. derived from electronic healthcare databases such as electronic medical records (EMR) databases or administrative health claims databases) or review of charts/records including medical record abstraction. The conduct, governance, and best practices for each are vastly different, and this paper will focus on secondary use of data studies only.

There is a growing body of literature on proposed guidelines for study design and methods for conducting high quality NIS [National Pharmaceutical Council (NPC)[1]; Strengthening the Reporting of Observational Studies in Epidemiology (STROBE)[2]; Agency for Healthcare Research and Quality (AHRQ)[3]; Patient-Centered Outcomes Research Institute (PCORI)[4]; European Network of Centers for Pharmacoepidemiology and Pharmacovigilance (ENCePP)[5]; International Society for Pharmacoepidemiology (ISPE, https://www.pharmacoepi.org/); International Society For Pharmacoeconomics and Outcomes Research (ISPOR, https://www.ispor.org/workpaper/practices_index.asp)[1]]. These guidelines provide advice on aspects to consider while designing a study and developing a protocol, but do not necessarily capture all aspects related to comprehensive best practices and common pitfalls to be avoided when implementing a NIS using secondary data sources from concept to publication. The intent of this paper is to propose a best practice for conducting secondary use of data NIS with the high level structure of starting with Concept, Protocol, Analysis Plan, Reporting and Dissemination, with some additional considerations.

## CONCEPT

A study concept is useful step in preparing to conduct a NIS. It is an opportunity to provide a high level description of the rationale, research questions, methods, data sources, and analyses before embarking on developing a full study protocol. The concept can be used to secure funding, organizational buy-in, support from external stakeholders, etc. before investing significant resources.

Common mistakes at this stage include: lack of a clear rationale, not understanding the advantages and limitations of the data source, not performing feasibility assessment, and lack of consideration of key operational elements.

A proposed NIS concept using secondary data sources should start with a clear rationale, highlighting what new insights will be learned, why such insights are important, and how the study might complement prior research. The rationale should feed nicely into well-defined research questions, which address an identified knowledge gap for the therapeutic area in question. Importantly, the research questions should build upon the current evidence base, and point to what evidence is lacking and thus requires validation.

A proper literature review should be conducted to identify prior research that can inform research questions, data sources, and study design. Similar research questions may have been addressed by other researchers. Therefore, before developing a full protocol it would be ideal to a) avoid duplication of a study that has already adequately addressed the research questions of interest; and b) identify limitations of prior research that can be improved upon, and learn from components of prior research that can be useful for the new study of interest.

Initial thinking on the study design should follow. This would include a description of the secondary data source(s) in adequate detail to demonstrate an understanding of the advantages and limitations regarding the population under study and availability of important variables. For example, a claims database used in the area of heart failure research typically does not include the New York Heart Association Functional Classification (NYHA) classification, an important risk factor and thus limits the usefulness of studies in this area. It is also important to have a data source that is relevant for the study design and with sufficient sample size.

A feasibility analysis can be useful to understand details of the data that is being analyzed. Analyses included at this preliminary stage include estimation of available sample size, variable distributions, variable (exposure and outcome) definitions, and data integrity (e.g. missingness, outliers). Before proceeding to a full protocol, it is important to ensure a sufficient sample will be available to address the research questions. This is true even for descriptive, non-hypothesis testing, studies, where estimates of precision should be used to determine if enough sample size is available to generate meaningful results.

In the sample size calculation, it is important to consider results from the literature, in terms of absolute or relative effects, effect sizes, variability, distributions, and put these in the context of findings from the feasibility. These can be used by an expert statistician or feed into sample size/power analysis software that allows for deriving power and sample size curves (e.g. PASS, https://www.ncss.com/software/). The calculated sample size for a pre-defined primary hypothesis (related to the study primary aim), and specified type I and type II error, are important aids in making go/no-go decision for different data sets or different design approaches that are evaluated within the study.

Finally, ensuring a well-rounded and qualified study team is in place, documentation of ethics committee review, and consideration of data accessibility and privacy are some key operational elements that should be considered at this early stage.

## PROTOCOL

### Objective

All efforts that are put into developing precise and clear objectives in the beginning of the study design will be rewarded during the subsequent phases of the project. Clear objectives will aid in identifying the appropriate study design, selection of data source, endpoint definition and in defining the appropriate statistical methods. Developing tightly-focused objectives defines how and what data is analyzed and provides a context for the results.

Common mistakes in articulating research objectives include: not having a single primary objective, not specifying the temporal parameters, and lack of specificity in the endpoint or outcome of the assessment. For example, a study objective 'to assess treatment patterns' is not specific enough to drive key study design and analysis parameters. A more specific 'treatment patterns' objective would be to assess the time to first line treatment among a specific cohort of patients. This would require a cohort to be defined by the presence of a diagnosis, but not yet treated, and then patients would be followed over time until first treatment occurs. Another possible 'treatment patterns' objective could be to assess treatment discontinuation rate. In this case, the cohort could be defined differently, and would include all treated patients followed until discontinuation. Similarly, an objective 'to assess burden of illness' is also not specific enough to define the outcomes of interest. A more specific 'burden of illness' objective would be to assess the frequency of healthcare resource utilization as measured by office visits, emergency room (ER) visits, and hospitalizations. Alternatively, one could assess the humanistic burden as measured by a disease specific quality of life instrument.

There can be several secondary objectives, however, there should only be one primary objective which should be aligned with the rationale, and is how methods, data sources, analyses, and sample size should be determined. It is also helpful when this protocol is written in a way that each objective can be evaluated throughout the protocol (study design, analysis, etc.).

Study objectives generally fall into the following two categories: descriptive and comparative (or analytic). The importance of the distinction in the type of objective is related to the amount of effort and attention given to maximizing the validity of each study.

Descriptive objectives are often used to understand patterns among variables, within a specified population and often are based on a host of personal characteristics (person, place, and time). Disease epidemiology studies, burden of illness studies, and the like, are typically descriptive. These studies often draw conclusions that can be generalized to a broader population and therefore external validity[6] is an important consideration.

From the variable patterns identified with descriptive objectives, researchers can develop hypotheses about the causes of these patterns and about the factors that may be associated with the occurrence of specific outcomes. Studies with descriptive objectives are often used to prepare or plan for studies with comparative (or analytic) objectives. The studies with comparative objectives will go a step further and quantitatively assess the relationship between two or more variables, and more specifically can statistically compare two or more groups on one or more endpoints or outcomes formally.

## Research Methods

Common mistakes in research methods include: not fully addressing all sources of potential bias; lack of clearly defined study population, identification period, study period and index date in study setting; lack of careful attention to the variables including coding differences across countries and data sources; lack of understanding of the data sources hence the generalizability of the results; and failure to address confidentiality, ethical considerations and adverse event reporting.

## Design

It is important to make sure that the study design and associated observation periods are well defined and appropriate for the research question. There are many different study designs that can be implemented when using secondary data sources. Some of the more common include: cross-sectional, (nested) case-control, retrospective cohort or case-crossover.

The design should capture key elements that will be implemented to minimize bias. Although some aspects of bias can be addressed in the analysis, it is often best to optimize the design to minimize different types of bias. There are several types of bias that can compromise the internal validity of a comparative analysis, which are well covered elsewhere.[7,8]

To avoid or minimize selection bias, efforts need to be made to ensure the control group represents the population of the investigated group, and loss to follow-up is minimized. Proper use of a control group allows the proper conclusion to be drawn in a comparative analysis study. Efforts should be made to match the demographic and baseline clinical characteristics of the control group to the "case" group. Certain methods such as propensity score matching or stratified matching can be used to achieve this goal. For example, to assess the difference of health care resource utilization as measured by office visits, emergency room visits and hospitalizations between multiple sclerosis (MS) group vs non-MS, the non-MS group should be matched with similar age, gender distribution, as well as other key baseline comorbidities. Restriction, matching and randomization methods are used at the design stage to minimize confounding bias. Randomization cannot be performed for secondary use of the data studies because the data has already been collected; however, restriction and matching both help minimize confounding. For example, restriction limits the study to people who are all in the same level or category of the potential confounder. In a study of multiple sclerosis patients, we may want to restrict the population to those that have disease activity with at least 1 or 2 relapses in a well-defined period. The advantages of this method are simple to control the confounder; while the disadvantages are lack of generalizability where the results may not apply to all relapse MS patients if we only study patients with active disease activity. Matching is used to select one or more controls that are similar to each case with respect to one or more potential confounding factors. The advantages are balance in measured confounders; however, the disadvantages are logistical difficulties in identifying suitable matched cohorts in the analysis.

**Setting**

The setting should provide details on the study population defined in terms of persons, place, study time period, and selection criteria, including the rationale for any exclusion criteria and their impact on the number of subjects available for analysis. Representativeness of the study population as regards the source population should be addressed. Where any sampling from a source population is undertaken, description of the source population and details of sampling methods should be provided.

A schematic of the study design is often helpful to describe the different time periods associated with the study. There is generally a 'study period' which describes the entire time period for the study including the earliest date a patient observation or healthcare encounter is used to the latest date a patient observation or healthcare encounter is used. An 'index event' date often identifies the event that makes a patient eligible for inclusion. All possible index events are identified from the 'identification period'. Depending on the overall design of the study, other periods may include a 'prior period' or 'follow-up period' where different study variables will be assessed relative to the index event date.

**Variables**

Careful attention to study variable definitions in secondary use of data studies is one of the most important aspects of non-interventional study implementation. For example, if one of the key variables of interest is not defined properly, then the entire study can be flawed, despite the best intentions with study design and use of sophisticated analytic methods. All study variables should be described in the protocol, considering, exposure, outcome, other analysis variables that could be used to address confounding or stratification, subgroups, etc.

In the case of a secondary use of data NIS, a best practice would be to start by considering the coding systems used by those that are populating the database to define the variables of interest. A critical and often overlooked exercise is for the study lead to sit down with a healthcare provider with experience in the therapeutic area and discuss patient pathways, pharmacotherapy and other care provided, and importantly address how the recording of diagnosis and care are implemented. Remember to also cover how these aspects have evolved over time, since the study is likely to be based on older data, if it is retrospective in nature. Having a basic understanding of the care environment where the study will take place, will help in the next step where one will need to consider how the end-points of the study should be defined. This process will comprise selecting variables, considering temporal aspects, and further evaluating proxies in the case that the design of choice is unlikely to support measuring the true end-point of interest. These aspects will be covered in more detail in the sections below.

Many different vocabularies exist for coding diagnoses and healthcare services such as procedures, labs, drugs, etc. These vocabularies vary across databases and across countries, making it very difficult to create consistent variable definitions across studies using different databases. Valuable on-line resources are available for the most commonly used systems, which can be queried against search terms for diseases and conditions, in order to provide comprehensive lists of codes.[9,10,11]

We recommend, however, leveraging information from prior studies (gleaned from literature reviews conducted at the concept stage) and the exercise described above of interviewing clinicians or conducting a chart review to understand how codes are actually implemented in practice. An important aspect of this exercise will also be to understand the level of detail that coding occurs, since quite frequently, codes can be very specific in theory, while in practice only general codes are used. This can be a deal-breaker if you want to study a specific condition, but the level of detail needed is rarely recorded. Let us use heart failure here as an example, where in 2016/2017 ICD-10-CM one would find a list of 17 unique ICD-10 codes that are found under the I50-chapter for heart failure. However, coding systems are often adapted to each country, and the I50-category can appear drastically different in a country-specific version, such as in the example of Sweden, where only two specific sub-codes are implemented (I50.1 and I50.9). If one were interested in specifically evaluating chronic heart failure, the ICD-10-CM list would give the idea that this level of detail is captured, while after having spoken to a physician, you might realize that the most common diagnosis code by far is I50.9. Furthermore, in an interview with clinicians, and using the example from above, one might find that less evident diagnosis codes (beyond the ICD-10 I50 chapter) are implemented to record patients with chronic heart failure, corresponding to e.g. cardiomyopathy. When in the process of selecting appropriate diagnosis codes to define the condition or outcome of interest in the study, evaluate whether these codes have evolved over time, and ensure that the code lists are contemporary to the data set. In addition, the diagnosis vocabulary of choice might also change over time, such as in the case of the US where the transition from ICD-9 to ICD-10 coding was implemented during 2015, creating a need for conversions between ICD-9 and ICD-10 in all studies with study periods that overlap the transition.

Given the limitations of medical coding vocabularies and the fact that most were not created for the purpose of doing research, it is often necessary to use variable proxies or to create algorithms. Also, the use of rule out diagnoses in clinical practice should be considered when thinking about variable definitions. A physician may record a heart failure diagnosis on a claim or encounter when performing a test of left ventricular ejection fraction, but if the test result is not indicative of heart failure (i.e., "rules out" HF), the use of that diagnosis code to include a patient in a heart failure cohort may introduce misclassification bias.

Further considering the chronic heart failure example, it is likely that heart failure severity may be an important covariate that may not be well recorded in a secondary data source. Some potential proxies for heart failure severity may be previous hospitalization, abnormal lab values, occurrence of a procedure, and prescriptions of drugs that can be specific for a certain condition or hint at a disease severity level. In the example of severe chronic heart failure above, a relevant suggestion could be to narrow the study population to patients that have a previous HF hospitalization, in the case that you only have access to diagnosis codes, and further to consider for example patients with NT-pro-BNP values ≥600 pg/mL, if the data source would have access to lab values.

Another example for creating an algorithm is in the case of identifying Multiple Sclerosis relapses. There is no specific code for MS relapses in US claims databases using ICD-9 CM diagnosis codes; however, Capkun *et al* used hospitalization visits, corticosteroid use, and timing between MS diagnoses to assess the occurrence relapse rates in different databases.[12]

**Data Sources**

When planning and implementing NIS, different data sources will have inherent characteristics that are important to consider when matching research objectives with data, and when considering generalizability of results. In addition to what was noted previously regarding variable definitions and availability, it is equally important to understand the advantages and limitations of the specific database selected for the research. As noted earlier, ideally, the objectives should be stated before identifying a data source, and not the other way around. Several key attributes that can impact whether a data source is applicable for addressing specific research questions include: setting of care (general practice, specialty, hospital, etc.), comprehensiveness of care (physician visits, hospitalizations, outpatient and inpatient pharmacy, labs, etc.), covered population (age, geographic variation, etc.). For example, the United Kingdom (UK) Clinical Practice Research Datalink (CPRD) is a primary care data based data source. Careful attention is required when trying to assess research questions relating to specialty care or the hospital setting because those data are collected separately, and must be explicitly requested and linked to the primary care data.

In Table 1 we have summarized some of the most common data sources, including inherent characteristics and caveats, such as care settings covered and of different data sources.

For the purposes of reporting the data source details in the protocol, the following attributes should be provided: Database name, brief database description, country/region, source of data, frequency of data collection/update, years covered, population description and size, sample weights (if applicable), description of key variable availability, coding vocabularies used, data validation, linkage to other databases (if applicable), description of database owner (e.g. government, commercial vendor), known use restrictions, references of other research done using the data.

**Table 1.** Common Data Sources

| Types of Databases | Description | Caveats |
|---|---|---|
| Health Insurance/Administrative claims databases | • Mainly collected to enable reimbursement of health costs by private or public insurers • Provides holistic view of reimbursed care provided for patient • Include basic demographics, physician and hospital care, procedures, and drug treatments and associated dates/costs • Allows for longitudinal study designs | • Population biased towards insured population for claim databases with private insurers (in US, employed population) • Limited lab results or biometry variables available, such as creatinine or BMI |
| Hospital/ Institutional databases | • These include central data repositories for an institution, such as an individual or group of hospitals and clinics. • The type and level of data incorporated in these databases are highly variable • Can capture specific information on e.g. lab values and details on in-hospital care • Can partly allow for longitudinal study designs | • Does not capture care sought outside of specified institution • For hospital databases, only specialized care is captured; is this relevant for research objective? |
| Electronic Health/ Medical Records (EHR/EMR) | • Contain information collected as part of routine medical care • Can provide a comprehensive view of patient medical history • Include basic demographics, physician and hospital care, procedures, and drug treatments and associated dates/costs • Lab and biometry variables available • Allows for longitudinal study designs | • Possible selection bias of contributors • Data limited to what is in the EHR/ EMR, so often lacking pharmacy fulfillment information • Loss of patient when they receive care by providers using different EMR system. • Lack of interoperability between EMR systems |
| Survey data | • Government- or third party-sponsored systematic healthcare surveys, conducted to assess public health, resource consumption, practice patterns and trends • Cross-sectional design | • Relies on patient-reported outcomes and associated recall-bias, needs to be taken into account for interpretation |
| Disease-specific registers | • A patient registry uses observational study methods to collect data on a particular patient population • Patient registries are often used to study the course of the disease and factors that affect outcomes • More detailed clinical information, compared to claims/EMR, in that disease-specific variables can be captured in a more structured and detailed format, e.g. symptom data (NYHA-class for HF), meta-information on drug treatment (e.g. up-titration, tolerability, daily dose, etc). • Cross-sectional design, limited longitudinally can be possible depending on design | • Possible selection bias of contributors • Does the register capture standard of care or rather best standard of care? • Often limited sample sizes |

## Protection of Human Subjects and Adverse Event (AE) Reporting

Proper data governance and protection of human subjects starts with ensuring that the rights, safety, and well-being of patients participating in non-interventional studies are protected (consistent with the principles that have their origin in the Declaration of Helsinki). It should not be assumed, that simply because a database exists and is available for research, that it can actually be used for the intended research.

The planning process should take into account whether approval from an independent ethics review board will be needed; whether informed consent of patients is required; how to ensure patient privacy, including potential anonymization procedures; and how data will be stored and handled. The considerations and laws to comply with can differ depending on where the study is conducted (local laws).

For studies based on secondary use of data, safety monitoring and safety reporting, where there is a safety relevant results, needs to be provided at an aggregate level only, and thus no reporting on an individual case is typically required. In studies based on secondary use of data with a safety relevant result, reports of adverse events/adverse reactions should be summarized in the study report, i.e. the overall association between an exposure and an outcome. Relevant findings from the study report will be included in the periodic aggregated regulatory reports submitted to Health Authorities.

## ANALYSIS PLAN

A statistical analysis plan (SAP) is recommended so that analysis details can be well documented and pre-specified. A common mistake that seems to occur often is the lack of pre-specified analysis, thereafter performing many ad-hoc and subgroup analyses to obtain the messages of interest. Pre-specified analysis will help in overcoming the criticism of cherry picking. In the SAP, details on the cohort definitions, variable definitions, and statistics that will allow any competent analyst to replicate the findings should be provided. The plan should provide detailed description of the statistical analysis for each objective.

The SAP should include the following: any changes to the analyses proposed in the protocol; details of the database version, dates, etc.; details of the analysis population; details of the study outcomes and other variables (including how missing data will be dealt with); statistical methods (including sensitivity analyses); and sample size estimation. One of the most critical components of an SAP is the table shells. It is important before any analyses begin that the proposed output tables are drafted, as this will help identify all variables that need to be reported, as well as metrics that will be used including N, %, mean/median, standard errors, confidence intervals, etc. An attrition table to show the targeted cohort extraction is recommended for most studies. Table 2 shows table shells for an attrition table, a summary table for continuous variables, and categorical variables.

**Table 2.** Example of Table Shells

**Patients Attrition Table**

| Criteria | Patients excluded | | Patients Remaining | |
|---|---|---|---|---|
| | n | % | n | % |
| Include patients had at least 1 non-ruleout MS diagnosis in identification period (10/1/2009 - 9/30/2015) | | | | |
| Include patients had at least 1 Gilenya drug claim during the identification period (10/1/2009 - 9/30/2015) | | | | |
| Include patients continuously enrolled in medical and pharmacy benefit 1 year prior to the index date | | | | |
| Include patients continuously enrolled in medical benefit and pharmacy 1 year post to the index date | | | | |
| Include patients age >= 18 and age<=65 at index date | | | | |

**Table 2. Example of Table Shells (continued)**

**Persistence by Region**

| **Characteristics** | **1 Year Persistence** | |
|---|---|---|
| | **n** | **%** |
| **Total Patients Completely Persistent:** | | |
| **By region** | | |
| Northeast | | |
| North Central | | |
| South | | |
| West | | |
| Unknown | | |

**Summary of Age at Index**

| **Characteristics** | **Gilenya Cohort (n=XX)** |
|---|---|
| **Age at Index** | |
| Mean | XX |
| 95% Confidence Interval | XX; XX |
| Standard Deviation | XX |
| Median | XX |
| IQR | XX; XX |
| Min; Max | XX; XX |

The SAP should provide a rationale for the choice of statistical techniques and major steps that lead from raw data to a final result, including methods used to correct inconsistencies or errors, impute values, modify raw data, categorize, analyze and present results, and procedures to control for sources of bias and their influence on results.

As noted earlier, many biases can be controlled with proper study design, however, sophisticated statistical analyses can also be applied to minimize or control/adjust for different types of bias. Details of these methods are beyond the scope of this paper, but can be found elsewhere.[1-5,13-15] The SAP should describe all statistical methods, including those used to control for confounding, to examine subgroups and interactions. For cohort studies explain how loss to follow up was addressed, if applicable. For case-control studies explain how matching of cases and controls was addressed, if applicable. For cross-sectional studies, describe analytical methods taking into account for sampling strategy, if applicable.

## REPORTING AND DISSEMINATION

Upon completion of the analyses, a study report should be prepared which provides a high level summary of what was done (excerpts from the protocol and analysis plan) and a summary of the results and conclusions. Summary results should be described in text with key tables and figures, starting with the study attrition showing the impact of all study design parameters including the inclusion and exclusion criteria (preferably in the form of a table or figure). Summary tables should be added as a supplement to the report summarizing all study findings. Additionally, electronic reports which include user-friendly and customizable visualizations may also supplement a written study report. This allows those interested to explore the study cohort to better understand different subgroups, sensitivity analyses, etc. Inferences about causal effects should be based on a variety of factors that should be explored in the discussion section with other limitations of the study. Investigators should not make inferences about causality based solely on the outcome of a test of significance (e.g., a p-value or a statement about the confidence interval including or not including the null value).

The decision to submit a NIS for peer reviewed publication should be made well in advance and should be documented in the protocol. All publications should comply with International Committee of Medical Journal Editors (ICMJE) guidelines.

## SUMMARY / CONCLUSION

In this paper, we have discussed common process of conducting a secondary use of data non-interventional study, from forming research questions and study objectives to study design and set up, and analysis consideration to study results dissemination. In many cases, we have also provided suggestions or accessible resources that researchers can put into practice. Summary of key considerations at each stage of the study are presented in Table 3.

**Table 3. Summary of Considerations for Designing Secondary Use of Data NIS**

| **Stage** | **Considerations** |
|---|---|
| Concept | • Have clear rationale<br>• Understand the advantages and limitations of the data source<br>• Perform feasibility assessment<br>• Consider key operational elements |
| Protocol | |
| 1) Objective | • Have single primary objective<br>• Specify the temporal parameters<br>• Clarify the specificity in the endpoint or outcome of the assessment<br>• Understand the difference between descriptive and comparative objective<br>• Consider generalizability and external validity<br>• Maximize internal validity to comparative study |
| 2) Research Methods | |
| Design | • Minimize bias |
| Setting | • Clearly define study population, study period and index date |
| Variables | • Pay attention to the variables including coding differences across countries and data sources |
| Data Sources | • Understand the data sources and the generalizability of the results |
| Ethics and AE | • Address confidentiality, ethical considerations and adverse event reporting |
| Analysis Plan | • Document and pre-specify analysis details |
| Reporting and Dissemination | • Document a high level summary of what was done and provide the results |

## Figures and Tables

**Table 1.** Common Data Sources

**Table 2.** Example of Table Shells

**Table 3.** Summary of Considerations for Designing Secondary Use of Data NIS

